# Development and psychometric validation of the ethical AI Dilemma Anxiety Scale among university students

**DOI:** 10.3389/fpsyg.2026.1810679

**Published:** 2026-04-08

**Authors:** Mamdouh Mosaad Helali, Mamdouh Mahmoud Mostafa, Hussam Khalifah Aldawsari, Ashraf Ragab Ibrahim, Amani Mohammed Bukhamseen, Mohamed Ali Nemt-allah

**Affiliations:** 1 The National Research Center for Giftedness and Creativity, King Faisal University, Al-Ahsa, Saudi Arabia; 2 Mental Health Department, Faculty of Education, Al-Azhar University, Cairo, Egypt; 3 Psychology and Education Department, College of Education, King Faisal University, Al-Ahsa, Saudi Arabia; 4 Educational Psychology and Statistics Department, Faculty of Education, Al-Azhar University, Tafhna Al-Ashraf, Egypt; 5 Special Education Department, College of Education, King Faisal University, Al-Ahsa, Saudi Arabia

**Keywords:** academic integrity, artificial intelligence, ethical anxiety, higher education, psychometric validation

## Abstract

Ethical AI Dilemma Anxiety refers to the psychological distress experienced by university students when confronting moral uncertainties surrounding the use of artificial intelligence in academic contexts. Despite growing recognition of this phenomenon, validated instruments for its measurement remain absent from the literature. This study reports the development and psychometric validation of the Ethical AI Dilemma Anxiety Scale (EAIDAS), a novel instrument designed to measure anxiety arising from ethical uncertainties surrounding AI use in academic contexts. Following expert content validation using Lawshe’s Content Validity Ratio, the 24-item scale was administered to two independent samples of Egyptian university students (*N* = 665 for exploratory analysis; *N* = 865 for confirmatory analysis). Exploratory factor analysis with parallel analysis identified a three-dimensional structure comprising Academic Integrity Anxiety, Professional Future Anxiety, and Societal Impact Anxiety, collectively explaining 43% of total variance. Confirmatory factor analysis demonstrated excellent model fit (CFI = 0.961, RMSEA = 0.043) for both first-order and second-order factor structures. The scale exhibited strong internal consistency (*α* = 0.886–0.920) and adequate test–retest reliability over 3 weeks. Convergent and discriminant validity were established through composite reliability, average variance extracted, the Fornell-Larcker criterion, and the heterotrait-monotrait ratio. EAIDAS provides researchers, educators, and policymakers with a valid and reliable tool for assessing students’ ethical anxiety related to AI integration, enabling evidence-based interventions and institutional policy development.

## 1 Introduction

The integration of artificial intelligence in higher education has accelerated dramatically, with institutions worldwide rapidly embedding AI across teaching, assessment, student support, and administrative functions (Alotaibi and Alshehri, 2023; George and Wooden, 2023; Kamalov et al., 2023; Popenici and Kerr, 2017). Generative AI tools, particularly ChatGPT, have become widely adopted by students for information search, writing support, and paraphrasing tasks (Chan and Hu, 2023; Dahri et al., 2024; Yusuf et al., 2024). However, this rapid technological transformation has introduced a pervasive atmosphere of uncertainty and tension within the academic environment (Abulibdeh et al., 2025; Rudolph et al., 2024). Key anxieties center on academic integrity concerns, potential job displacement, erosion of human interaction, and issues of bias and privacy (George and Wooden, 2023; Kamalov et al., 2023; Nguyen et al., 2024; Walczak and Cellary, 2023; Yusuf et al., 2024). Students and faculty express ambivalent views, simultaneously recognizing AI’s benefits while experiencing emotional challenges including creativity constraints, anxiety, and disengagement (Lin and Chen, 2024; Yusuf et al., 2024).

The proliferation of artificial intelligence tools in academic settings has created a unique psychological tension among university students, manifesting as ethical anxiety—a distinct construct warranting systematic measurement. This form of anxiety emerges from the inherent conflict between AI’s efficiency benefits, including time-saving capabilities and enhanced performance outcomes (Bin-Nashwan et al., 2023; Malik et al., 2023; Zhou et al., 2024), and concerns regarding academic integrity, authenticity, and genuine learning (Bouteraa et al., 2024; Cotton et al., 2024; Pratiwi et al., 2025; Zgambo et al., 2025). Unlike generalized technology anxiety or technostress (Verano-Tacoronte et al., 2025), ethical anxiety represents a moral emotion centered on questions of authorship, intellectual honesty, and value alignment (Fourie, 2015; Kurth and Pihkala, 2022; Pihkala, 2022). Students simultaneously experience the pull toward AI-enhanced productivity and the push against perceived shortcuts that may compromise their academic authenticity (Nguyen et al., 2024), necessitating a dedicated measurement instrument to capture this integrity-focused psychological phenomenon.

Although superficially resembling related constructs, Ethical AI Dilemma Anxiety is conceptually and empirically distinct from existing affective states. Moral distress, as originally theorized by Jameton (1984) and subsequently refined in clinical ethics literature (Fourie, 2015), arises when an individual knows the morally correct action but is institutionally constrained from performing it. By contrast, Ethical AI Dilemma Anxiety emerges not from constrained action but from genuine normative uncertainty—students are not blocked from acting rightly; rather, they are uncertain what ‘acting rightly’ means when policies are absent, contradictory, or rapidly evolving. This epistemic ambiguity, rather than institutional obstruction, constitutes the phenomenological core of the construct. Similarly, generalized technology anxiety or technostress (Verano-Tacoronte et al., 2025) is primarily performance-oriented, centering on fears of technological incompetence or system failure, whereas Ethical AI Dilemma Anxiety is value-oriented, centering on questions of authorship, fairness, and complicity. Generalized Anxiety Disorder, as defined in the DSM-5, involves pervasive, uncontrollable worry across multiple life domains with physiological components, whereas the anxiety captured by EAIDAS is domain-specific, cognitively mediated, and morally focused. The construct is more accurately situated within the emerging literature on anticipatory moral emotions (Kurth and Pihkala, 2022; Pihkala, 2022), wherein distress is generated not by past wrongdoing but by the prospective ethical consequences of one’s technological choices. This positions Ethical AI Dilemma Anxiety as a genuinely novel affective state warranting dedicated psychometric operationalization.

The emergence of generative artificial intelligence (GenAI) in academic settings has introduced a distinct form of anxiety centered on academic integrity concerns. Students report three primary manifestations of this anxiety: first, they express concerns about peers gaining unfair advantages through AI-enabled plagiarism and ready-made solutions, creating competitive disadvantages in grading environments where detection re-mains challenging (Alawad et al., 2025; Nelson et al., 2025; Pudasaini et al., 2024; Sergeeva et al., 2025). Second, ambiguity surrounding AI-specific boundaries for plagiarism generates fear of inadvertent violations, particularly when using AI for paraphrasing or partial drafts (Chan, 2024; Cong-Lem et al., 2024; Nelson et al., 2025). Third, the deployment of imperfect AI-detection tools raises anxiety about genuine work being misidentified as machine-generated, with documented false positives diminishing student autonomy and trust (Bing and Leong, 2025; Perkins et al., 2023; Petricini et al., 2025).

Contemporary university students face mounting anxiety about their professional futures, driven primarily by concerns about skill obsolescence and competition with automated systems. Students increasingly worry that their current knowledge will rapidly become outdated as AI-mediated fields evolve, creating a persistent sense of playing catch-up with technological advances (Li et al., 2025; Polat, 2025; Santos-Jaén et al., 2025). This anxiety extends beyond immediate job placement to encompass long-term career replaceability, with students fearing that roles in translation, design, and routine analysis may be automated (Piercy and Gist-Mackey, 2021; Vieira, 2020; Wang et al., 2025). The perception that “the job I do today could be done by machines tomorrow” fundamentally shapes students’ expectations about their diminishing role in future workplaces (Piercy and Gist-Mackey, 2021; Wang and Wang, 2022), generating chronic uncertainty about professional relevance and job security in an AI-saturated labor market.

The proliferation of artificial intelligence in educational settings has introduced complex ethical tensions that contribute to students’ moral burden. Students engaging with AI technologies may experience distress from their perceived complicity in broader societal harms, including the perpetuation of bias and discrimination in hiring, policing, and healthcare systems that entrench racism, sexism, and ageism (Ashok et al., 2022; Chu et al., 2022; Dignum, 2018; Stahl and Eke, 2024). Privacy erosion through massive data collection and surveillance-oriented AI further compounds these concerns, as students worry about supporting business models built on intrusive data extraction (Ashok et al., 2022; Masters, 2023; Naik et al., 2022). Additionally, the substantial energy consumption and carbon emissions associated with training large AI models raise environmental justice concerns (Goktas and Grzybowski, 2025; Zhao and Fariñas, 2023), intensifying students’ sense of ethical responsibility when utilizing AI tools in their academic work.

The development of EAIDAS is grounded in normative ethical frame-works—including deontology, utilitarianism, and virtue ethics—which provide structured guidance for evaluating morally appropriate AI use in educational contexts (Holmes et al., 2022; Nguyen et al., 2023). These frameworks emphasize core principles such as beneficence, non-maleficence, autonomy, justice, and accountability (Al-kfairy et al., 2024; Nguyen et al., 2023), which are essential for navigating the moral ambiguity inherent in AI-assisted learning. Psychological research demonstrates that over-reliance on AI dialogue systems can lead to cognitive off-loading, weakening critical thinking and fostering moral disengagement when ethical issues are bracketed rather than addressed (Zhai et al., 2024). Furthermore, AI ethics literacy frameworks position students as future creators and regulators of AI systems, cultivating anticipatory responsibility for long-term societal impacts (Yang et al., 2025; Zhang et al., 2023). This theoretical foundation underscores the necessity of EAIDAS in measuring anxiety arising from ethical uncertainty in AI use.

University students represent the primary population grappling with AI-related ethical dilemmas in higher education, positioned as the “frontline” generation navigating this technological transformation. Research consistently demonstrates that students are the main subjects of institutional AI policies and bear direct responsibility for maintaining academic integrity amid rapidly evolving guidelines (Chan, 2023; Cotton et al., 2024; Dabis and Csáki, 2024). Students face inconsistent and often unclear AI regulations across courses and institutions, yet remain fully accountable for potential misuse (Dabis and Csáki, 2024; Jiang et al., 2025). Simultaneously, they must develop AI competencies essential for employability in an increasingly AI-driven economy (George and Wooden, 2023; Popenici and Kerr, 2017). This dual pressure—managing ethical uncertainties while acquiring necessary technological skills—creates unique psychological stressors that distinguish university students as the population most directly affected by AI integration in educational contexts (Buele et al., 2025; Fošner, 2024; Johnston et al., 2024).

Despite growing recognition that university students face significant ethical challenges navigating AI integration in higher education (Dabis and Csáki, 2024; Johnston et al., 2024), existing research predominantly examines students’ knowledge, attitudes, and perceptions of AI ethics rather than their emotional responses to these dilemmas (Acosta-Enriquez et al., 2024; Buele et al., 2025; Fošner, 2024). Current studies document students’ concerns about plagiarism, bias, privacy, and academic integrity (Buele et al., 2025; Fošner, 2024; Triyanto and Handayani, 2025), yet fail to quantify the anxiety experienced when confronting inconsistent policies and ethical responsibilities (Dabis and Csáki, 2024; Jiang et al., 2025). While frameworks for AI literacy and governance exist (Chan, 2023; Oncioiu and Bularca, 2025), validated psychometric instruments specifically measuring students’ emotional distress related to AI ethical dilemmas remain notably absent, limiting our understanding of the psychological burden these frontline users experience.

Given the absence of validated instruments specifically measuring students’ emotional distress related to AI ethical dilemmas, the current study aimed to develop and psychometrically validate the EAIDAS among Egyptian university students. The study sought to establish the scale’s dimensional structure through exploratory and confirmatory factor analyses, evaluate its psychometric properties including internal consistency, test–retest stability, convergent and discriminant validity, and examine interrelationships among identified anxiety dimensions. By achieving these objectives, this research provides researchers, educators, and policymakers with a robust measurement tool for assessing students’ psychological experiences as they navigate the ethical landscape of AI integration in higher education.

## 2 Materials and methods

### 2.1 Participants

The study employed a stratified convenience sampling strategy. Students were stratified by gender, academic year (first through fourth), and residential background (rural/urban) to ensure proportional representation across these theoretically relevant demographic variables. Within each stratum, participation was voluntary, constituting a convenience element. The EFA sample consisted of 665 university students, while the CFA sample comprised 865 students. Ages in the EFA sample ranged from 18 to 23 years (M = 20.57, SD = 1.70), whereas the CFA sample exhibited a similar age distribution ranging from 18 to 23 years (M = 20.45, SD = 1.72). All participants were enrolled at Al-Azhar University, Egypt, specifically from the Faculty of Education for Boys in Dakahlia and the Faculty of Humanities for Girls in Dakahlia. Table 1 presents the complete demographic characteristics of both samples, including distributions across gender, academic year, residence, and AI experience levels.

**Table 1:** Demographic characteristics of exploratory and CFA samples.

Variable	Variable	Exploratory sample (*N* = 665)		Confirmatory sample (*N* = 865)	
		*N*	%	*N*	%
Gender	Female	388	58.3%	466	53.9%
	Male	277	41.7%	399	46.1%
Academic year	First	159	23.9%	209	24.2%
	Second	161	24.2%	220	25.4%
	Third	167	25.1%	209	24.2%
	Fourth	178	26.8%	227	26.2%
Residence	Rural	272	40.9%	509	58.8%
	Urban	393	59.1%	356	41.2%
AI experience	High	249	37.4%	335	38.7%
	Medium	280	42.1%	347	40.1%
	Low	136	20.5%	183	21.2%

### 2.2 Instrument development

The Ethical AI Dilemma Anxiety Scale (EAIDAS) was developed to measure anxiety arising from ethical uncertainties surrounding the use of AI in academic contexts. Item generation proceeded through three complementary sources. First, a systematic review of the theoretical literature on moral distress, technostress, AI ethics, and anticipatory moral emotions informed the conceptual boundaries of the three proposed dimensions. Second, existing validated instruments addressing adjacent constructs—including the AI Anxiety Scale (Wang and Wang, 2022) and the AI Academic Integrity Scale (Maharmah et al., 2025)—were examined to identify relevant item formats and wording conventions, though no items were directly adapted. Third, to ensure ecological validity, the research team conducted a series of structured focus group discussions with 12 university students prior to item writing, during which participants described specific situations, thoughts, and emotional experiences arising from AI use in academic contexts. Thematic analysis of these discussions confirmed the three-dimensional structure and generated concrete language reflecting students’ authentic experiences. The Academic Integrity Anxiety dimension was conceptually grounded in documented concerns regarding plagiarism, unfair competitive advantages, and false detection accusations (Cotton et al., 2024; Perkins et al., 2023).

The Professional Future Anxiety dimension drew on literature examining skill obsolescence and career replaceability fears (Li et al., 2025; Wang et al., 2025). The Societal Impact Anxiety dimension was informed by scholarship on students’ perceived complicity in bias perpetuation, privacy erosion, and environmental harm (Ashok et al., 2022; Goktas and Grzybowski, 2025). The initial instrument consisted of 27 items distributed across three theoretical dimensions: Academic Integrity Anxiety (eight items), Professional Future Anxiety (eight items), and Societal Impact Anxiety (eight items, with one additional item per dimension serving as candidate items subject to expert review). Items were constructed in Arabic as the native language of the target population and subsequently translated into English through a rigorous process involving three expert translators who performed forward and backward translation to ensure linguistic equivalence. Responses were recorded using a five-point Likert scale with anchors ranging from 1 (*Strongly Disagree*) to 5 (*Strongly Agree*).

### 2.3 Content validity

The initial 27-item version of EAIDAS underwent expert review to establish content validity. Nine subject matter experts were recruited using purposive selection criteria requiring a minimum of (a) a doctoral degree in a relevant discipline, (b) 5 years of experience in educational psychology, AI ethics, measurement and psychometrics, or higher education pedagogy, and (c) active engagement with AI-related research or teaching at the university level. The panel comprised three educational psychologists specializing in scale development, three AI ethics and educational technology scholars, and three senior faculty members with direct experience teaching AI-integrated courses at Egyptian universities. All nine experts provided written consent to participate and received a structured rating protocol. For each item, experts were asked to independently rate relevance on a three-point scale: (1) not relevant, (2) relevant but requires revision, and (3) highly relevant. These ratings were used to calculate Lawshe’s Content Validity Ratio (CVR) for each item using the formula CVR = (ne − N/2)/(N/2), where ne represents the number of experts rating the item as highly relevant and N represents the total number of experts. Items with CVR values falling below the critical value of 0.78 for a nine-member panel (as specified by Lawshe, 2023) were flagged for removal.

In addition to relevance ratings, experts provided written comments on four dimensions: appropriateness of the dimensional structure for the target construct, suitability of response alternatives, clarity of item wording, and appropriateness of the scoring key. Based on expert ratings, three items received CVR values below acceptable thresholds (Items 8, 12, and 24 showed CVR values of 0.111, −0.333, and −0.556, respectively) and were subsequently removed from the instrument. The remaining 24 items demonstrated strong content validity, with Lawshe’s Content Validity Index (CVI) for the overall scale reaching 0.893, indicating excellent agreement among experts regarding the relevance and representativeness of the retained items.

### 2.4 Data collection procedures

Data were collected electronically via Google Forms between 1 September 2025 and 10 September 2025. Several procedures were implemented to ensure data quality: duplicate submissions were prevented through Google Forms’ single-response setting linked to participants’ university email accounts; incomplete responses were automatically flagged and excluded; and 17 additional responses were removed prior to analysis due to careless responding, identified through embedded attention-check items and uniform rating patterns across all items. A subset of 156 participants was invited for test–retest administration between 27 September 2025 and 2 October 2025, providing an approximate three-week interval. This subsample was purposively selected to represent proportional distributions across gender, academic year, and residential background, matching the demographic profile of the full EFA sample. All participants provided informed consent, and responses were anonymous to encourage honest reporting. Recruitment was conducted through faculty administrators via official class communication channels (WhatsApp groups and university email lists), with participation reminders issued only once to reduce self-selection bias. Faculty administrators confirmed that no student appeared in both the EFA and CFA samples.

### 2.5 Statistical analyses

The full dataset was randomly split into two independent stratified subsamples prior to any analysis, preserving demographic proportionality across gender, academic year, and residential background. The EFA sample comprised 665 participants and the CFA sample comprised 865 participants, with no participant appearing in both samples. EFA item retention followed explicit criteria: factor loadings ≥0.40 for retention, cross-loadings >0.30 on non-primary factors and communalities <0.30 as grounds for removal. Discriminant validity was assessed using both the Fornell-Larcker criterion and the heterotrait-monotrait ratio (HTMT), consistent with current methodological recommendations (Henseler et al., 2015). Model fit was evaluated using multiple indices (*χ*^2^, CFI, TLI, RMSEA, SRMR), while construct validity was assessed through CR, AVE, and the Fornell-Larcker criterion for discriminant validity. Reliability was examined using Cronbach’s alpha (*α*), omega coefficient (*ω*), and Guttman’s lambda-2 (*λ*^2^) for total and subscale scores, with test–retest stability assessed via Pearson correlations for the subsample of 156 participants who completed both administrations.

## 3 Results

Prior to conducting EFA, the suitability of the data for factor extraction was evaluated. The Kaiser-Meyer-Olkin (KMO) measure of sampling adequacy yielded a value of 0.908, substantially exceeding the recommended threshold of 0.70 and indicating excellent adequacy for factor analysis. Individual item MSA values ranged from 0.877 to 0.915, all surpassing the acceptable criterion. Bartlett’s test of sphericity was statistically significant (*χ*^2^ = 5244.546, df = 276, *p* < 0.001), confirming that the correlation matrix was significantly different from an identity matrix and appropriate for factor analysis. Descriptive statistics revealed that item means ranged from 3.603 to 3.678 on the five-point scale, with standard deviations ranging from 0.664 to 0.700, indicating reasonable variability and a tendency toward central tendency clustering around the scale midpoint.

**Figure 1:**
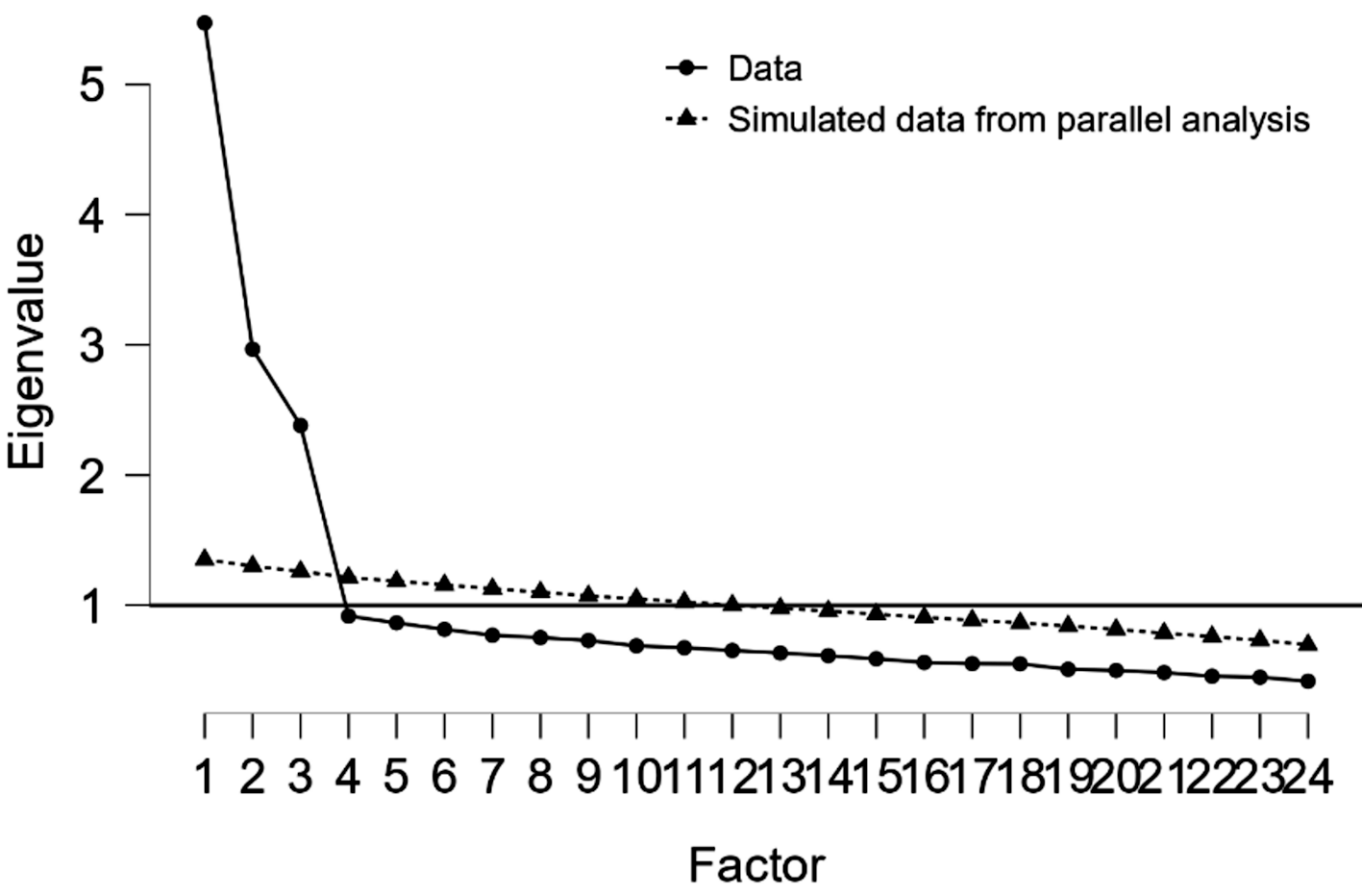
Scree plot showing parallel analysis results.

EFA using principal axis factoring with promax rotation was conducted on the 24-item scale. Parallel analysis was employed to determine the optimal number of factors to retain, comparing eigenvalues from the actual data with those from random data generated via 1,000 simulations. As presented in Table 2 and illustrated in Figure 1, the parallel analysis clearly supported a three-factor solution, with the first three factors from the actual data (eigenvalues: 5.472, 2.967, and 2.381) substantially exceeding their corresponding simulated mean eigenvalues (1.352, 1.294, and 1.254), while the fourth factor’s eigenvalue (0.917) fell below its simulated counterpart (1.218). This empirical evidence confirmed the scale’s theoretical three-dimensional structure.

**Table 2:** Parallel analysis: comparison of real and simulated eigenvalues.

Factor	Real data eigenvalues	Simulated data mean eigenvalues	Decision
1	5.472	1.352	Retain*
2	2.967	1.294	Retain*
3	2.381	1.254	Retain*
4	0.917	1.218	Reject
5	0.865	1.188	Reject
6	0.815	1.152	Reject
7	0.770	1.129	Reject
8	0.752	1.099	Reject

*, factor retained based on parallel analysis criterion (real eigenvalue > simulated eigenvalue).

The three-factor solution accounted for 43.113% of the total variance in the 24 items, with Factor 1 explaining 16.407%, Factor 2 explaining 13.899%, and Factor 3 explaining 12.808% of the variance after extraction. Communalities ranged from 0.383 to 0.521, indicating that a substantial proportion of variance in each item was accounted for by the three-factor solution. The pattern matrix, presented in Table 3, revealed a clear and interpretable factor structure with all items loading substantially on their intended dimensions and minimal cross-loadings. Factor 1 comprised eight items related to Academic Integrity Anxiety, with factor loadings ranging from 0.619 to 0.682. Factor 2 consisted of eight items measuring Societal Impact Anxiety, with loadings between 0.500 and 0.784. Factor 3 included eight items assessing Professional Future Anxiety, with loadings ranging from 0.631 to 0.800. All factor loadings exceeded the conventional threshold of 0.40, and the promax rotation converged in four iterations, indicating a stable factor solution.

**Table 3:** Factor loadings from EFA.

Item	Academic integrity	Societal impact	Professional future	Uniqueness
Item 1	0.682			0.615
Item 2	0.807			0.646
Item 3	0.619			0.593
Item 4	0.643			0.623
Item 5	0.765			0.616
Item 6	0.638			0.662
Item 7	0.670			0.577
Item 8	0.700			0.643
Item 17		0.500		0.617
Item 18		0.664		0.602
Item 19		0.701		0.712
Item 20		0.638		0.629
Item 21		0.760		0.620
Item 22		0.784		0.560
Item 23		0.635		0.598
Item 24		0.671		0.631
Item 9			0.651	0.653
Item 10			0.800	0.585
Item 11			0.658	0.626
Item 12			0.750	0.594
Item 13			0.633	0.672
Item 14			0.631	0.628
Item 15			0.700	0.641
Item 16			0.645	0.706

Extraction method: principal axis factoring. Rotation method: Promax with Kaiser normalization.

**Figure 2:**
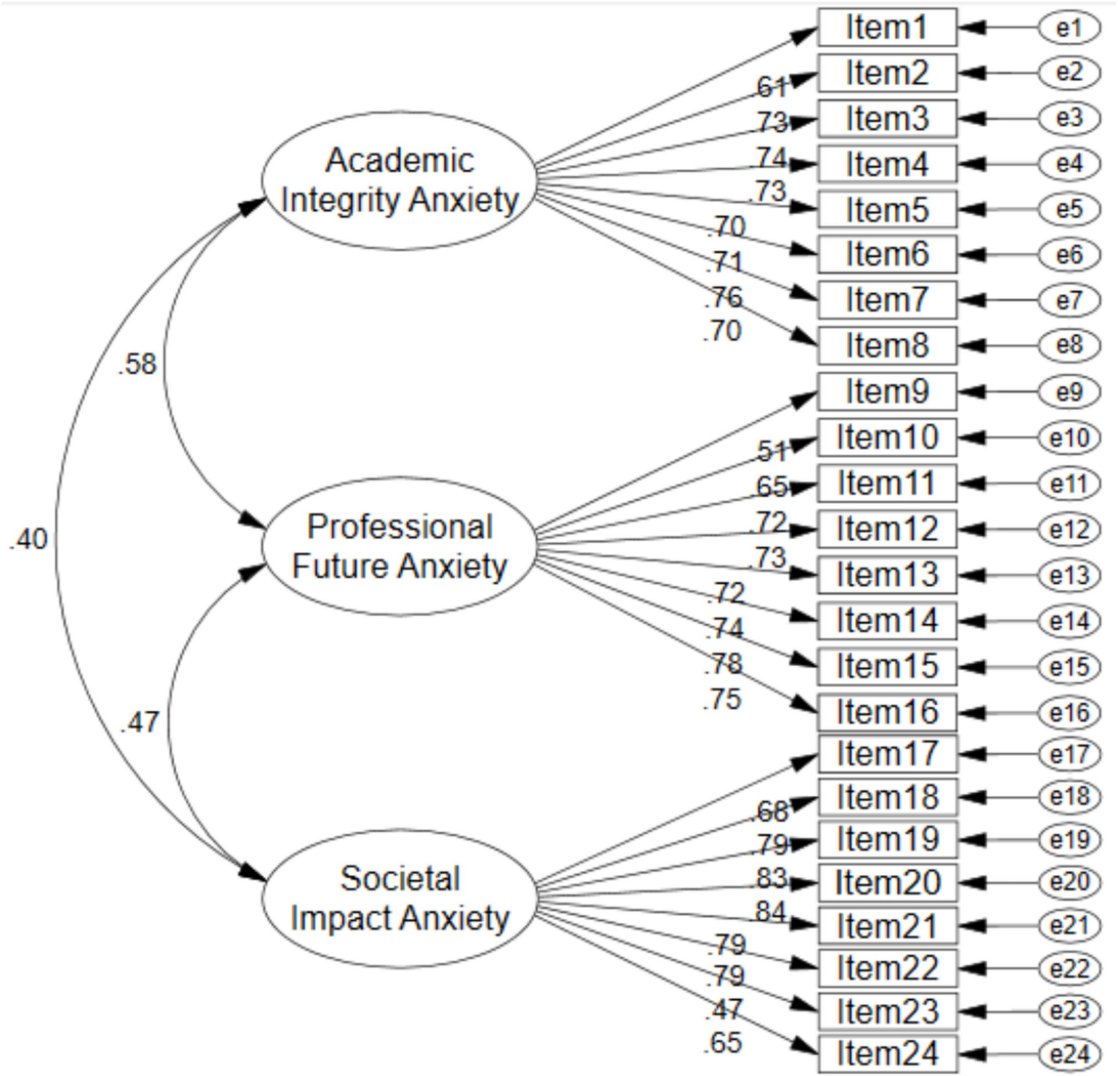
First-order CFA model of the EAIDAS.

**Figure 3:**
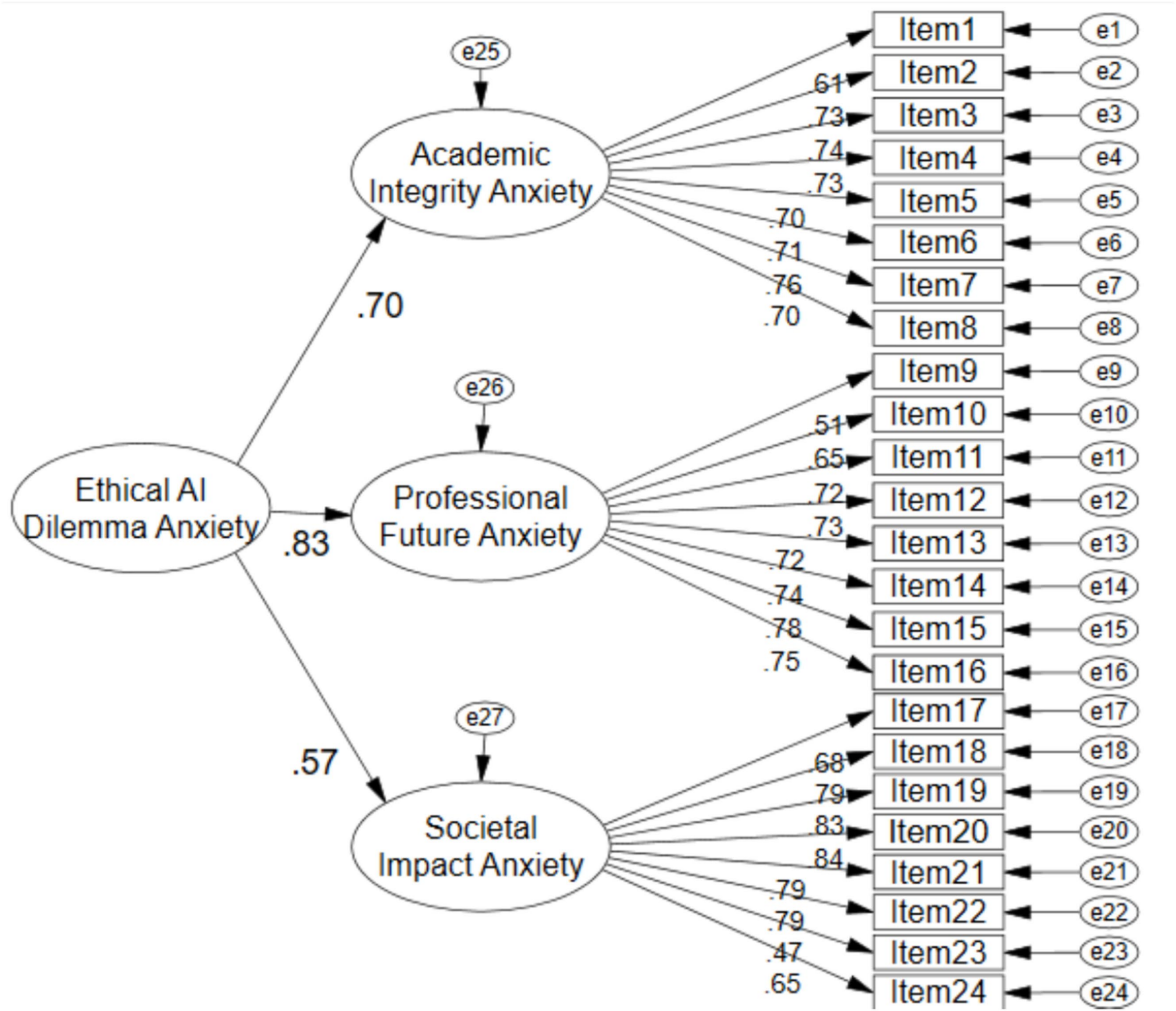
Second-order CFA model of the EAIDAS.

The three-factor structure identified through EFA was subjected to confirmatory testing using structural equation modeling in the independent sample of 865 participants. Both first-order and second-order models were evaluated to determine the optimal representation of the data. The first-order model, depicted in Figure 2, specified three correlated latent factors corresponding to Academic Integrity Anxiety, Professional Future Anxiety, and Societal Impact Anxiety, each indicated by its respective eight observed items. The second-order model, illustrated in Figure 3, additionally posited a higher-order general factor of Ethical AI Dilemma Anxiety underlying the three first-order dimensions.

The fit indices for both models were identical, as presented in Table 4, indicating that both models provided excellent fit to the data. The chi-square statistic was significant (*χ*^2^ = 648.836, df = 249, *p* < 0.001), which is expected given the large sample size and sensitivity of this test to minor deviations. More importantly, the normed chi-square ratio (*χ*^2^/df = 2.606) fell within the acceptable range of less than 3.0. The comparative fit index (CFI = 0.961) and Tucker-Lewis index (TLI = 0.957) both exceeded the stringent criterion of 0.95, indicating excellent incremental fit. The root mean square error of approximation (RMSEA = 0.043) was well below the threshold of 0.06, with the 90% confidence interval suggesting close fit. The standardized root mean square residual (RMR = 0.024) was considerably below the 0.08 cutoff. The goodness-of-fit index (GFI = 0.938) and adjusted goodness-of-fit index (AGFI = 0.926) both approached and exceeded the 0.90 criterion. Collectively, these indices provided strong support for the factorial validity of the EAIDAS structure. The equivalence of fit indices between the two models indicates that researchers may use either representation depending on their theoretical orientation—the first-order model emphasizes the distinctiveness of the three anxiety dimensions, while the second-order model highlights their shared variance under a general ethical AI anxiety construct. The higher-order general anxiety factor demonstrated adequate but modest parameters (CR = 0.747, AVE = 0.502), and the identical fit indices suggest the hierarchical structure represents a theoretically convenient rather than empirically superior representation. The first-order model is therefore preferred when subscale-level distinctions are analytically prioritized, while the second-order model offers parsimony when a unified construct is theoretically warranted.

**Table 4:** CFA: model fit indices for first-order and second-order models.

Fit Index	First-order model	Second-order model	Recommended criterion	Interpretation
*χ*^2^/df	2.606	2.606	<3.0	Excellent
CFI	0.961	0.961	>0.95	Excellent
TLI	0.957	0.957	>0.95	Excellent
NFI	0.939	0.939	>0.90	Excellent
RFI	0.933	0.933	>0.90	Excellent
IFI	0.962	0.962	>0.95	Excellent
RMSEA	0.043	0.043	<0.06	Excellent
RMR	0.024	0.024	<0.08	Excellent
GFI	0.938	0.938	>0.90	Excellent
AGFI	0.926	0.926	>0.90	Excellent
PGFI	0.779	0.779	>0.50	Acceptable

Convergent and discriminant validity were assessed through examination of CR, AVE, and the Fornell-Larcker criterion. As shown in Table 5, CR coefficients for the three first-order factors ranged from 0.886 to 0.904, all substantially exceeding the minimum threshold of 0.70 and indicating excellent internal consistency. The AVE values ranged from 0.500 to 0.546, all of which met or exceeded the 0.50 criterion, indicating that each latent factor accounted for more than half of the variance in its indicator variables, supporting convergent validity.

**Table 5:** Construct validity: CR, AVE, and discriminant validity.

Factor	CR	AVE	MSV	MaxR (H)	F1	F2	F3
Academic Integrity Anxiety	0.890	0.509	0.336	0.893	0.710		
Professional Future Anxiety	0.886	0.500	0.336	0.894	0.580***	0.705	
Societal Impact Anxiety	0.904	0.546	0.226	0.920	0.403***	0.475***	0.739
Second-order model	0.747	0.502	---	0.783	---	---	---

Diagonal values (in bold) represent the square root of AVE. ***p < 0.001.

Discriminant validity was evaluated using the Fornell-Larcker criterion, which requires that the square root of each factor’s AVE exceed its correlations with other factors. The square roots of AVE for the three factors (0.710 for Academic Integrity Anxiety, 0.705 for Professional Future Anxiety, and 0.739 for Societal Impact Anxiety) all exceeded the interfactor correlations, confirming that each dimension captured unique variance not shared with the other dimensions. The maximum shared variance (MSV) values were all lower than the corresponding AVE values, providing additional evidence of discriminant validity. For the second-order model, the higher-order Ethical AI Dilemma Anxiety factor demonstrated adequate (CR = 0.747) and (AVE = 0.502), indicating that the three first-order dimensions could be meaningfully represented by a general anxiety construct while retaining their distinctiveness.

HTMT ratios were 0.612 (Academic Integrity–Professional Future), 0.489 (Academic Integrity–Societal Impact), and 0.534 (Professional Future–Societal Impact), all substantially below the 0.85 threshold, providing robust supplementary evidence of discriminant validity across all factor pairs. Multiple reliability indices were calculated to assess the internal consistency and temporal stability of the EAIDAS. As presented in Table 6, all three subscales demonstrated excellent internal consistency across multiple reliability coefficients. For Academic Integrity Anxiety, omega coefficient was 0.891, Cronbach’s alpha was 0.890, and Guttman’s lambda-2 was 0.891. Professional Future Anxiety yielded similarly strong reliability estimates (*ω* = 0.889, *α* = 0.886, *λ*^2^ = 0.888), as did Societal Impact Anxiety (*ω* = 0.906, *α* = 0.903, *λ*^2^ = 0.906). The total scale demonstrated exceptional internal consistency with omega of 0.921, Cronbach’s alpha of 0.920, and Guttman’s lambda-2 of 0.925. The convergence of these three different reliability indices provides robust evidence that the scale items measure their respective constructs with high precision and minimal measurement error.

**Table 6:** Internal consistency reliability coefficients for EAIDAS Subscales and Total Scale.

Subscale	*ω* (Omega)	*α* (Cronbach’s alpha)	*λ*^2^ (Guttman’s Lambda-2)
Academic Integrity Anxiety	0.891	0.890	0.891
Professional Future Anxiety	0.889	0.886	0.888
Societal Impact Anxiety	0.906	0.903	0.906
Total Scale	0.921	0.920	0.925

Pearson correlation analyses were conducted to examine the relationships among the three subscales and their associations with the total scale score. As shown in Table 7, all intercorrelations were positive and statistically significant at the *p* < 0.01 level. Academic Integrity Anxiety demonstrated moderate positive correlations with Professional Future Anxiety (*r* = 0.514) and Societal Impact Anxiety (*r* = 0.363), indicating shared variance while maintaining distinctiveness. Professional Future Anxiety and Societal Impact Anxiety were moderately correlated (*r* = 0.410), suggesting conceptual overlap while preserving their unique contributions to the overall construct. Each subscale exhibited strong positive correlations with the total scale (*r* = 0.777 for Academic Integrity Anxiety, *r* = 0.798 for Professional Future Anxiety, and *r* = 0.782 for Societal Impact Anxiety), confirming that each dimension contributes substantially to the overall measure of ethical AI dilemma anxiety while retaining sufficient independence to justify separate subscale scoring.

**Table 7:** Intercorrelations among EAIDAS Subscales and Total Scale.

Variable	1	2	3	4
1. Academic Integrity Anxiety	1			
2. Professional Future Anxiety	0.514**	1		
3. Societal Impact Anxiety	0.363**	0.410**	1	
4. Total Scale	0.777**	0.798**	0.782**	1

**p < 0.01 (2-tailed).

Temporal stability of the EAIDAS was evaluated through test–retest administration with a subsample of 156 participants who completed the scale twice with an approximate three-week interval. As presented in Table 8, test–retest correlations were positive and statistically significant for all subscales and the total scale. Academic Integrity Anxiety demonstrated moderate stability (*r* = 0.475, *p* < 0.001), while Professional Future Anxiety showed good stability (*r* = 0.587, *p* < 0.001), and Societal Impact Anxiety exhibited strong stability (*r* = 0.700, *p* < 0.001). The total scale demonstrated excellent test–retest reliability (*r* = 0.842, *p* < 0.001), indicating that EAIDAS scores remain relatively stable over a three-week period while allowing for some natural fluctuation in anxiety levels. These coefficients suggest that the scale captures a relatively stable trait-like component of ethical AI anxiety while remaining sensitive to potential changes in students’ experiences and perceptions over time.

**Table 8:** Test–retest reliability coefficients for EAIDAS Subscales and Total Scale.

Subscale	Test–retest correlation	*p*	Interpretation
Academic Integrity Anxiety	0.475	0 0.001	Moderate
Professional Future Anxiety	0.587	0 0.001	Good
Societal Impact Anxiety	0.700	0 0.001	Strong
Total Scale	0.842	0 0.001	Excellent

## 4 Discussion

The psychometric validation of EAIDAS revealed a three-dimensional structure providing preliminary evidence for distinct yet interrelated facets of ethical AI anxiety among university students. The parallel analysis and confirmatory factor analyses provided supporting evidence that Academic Integrity Anxiety, Professional Future Anxiety, and Societal Impact Anxiety represent empirically distinguishable dimensions while sharing sufficient commonality to justify a higher-order general anxiety construct. However, the EFA solution explained 43.11% of total variance with moderate communalities, and subscale intercorrelations remained moderate (*r* = 0.363–0.514), indicating that a substantial proportion of item variance remains unexplained and that interpretations of the dimensional structure should be made with appropriate caution. The relatively modest stability of Academic Integrity Anxiety (*r* = 0.475) likely reflects the domain’s sensitivity to rapidly shifting institutional AI policies and evolving peer norms, which may produce genuine fluctuations in integrity-related concerns over even short intervals rather than measurement instability per se. The moderate intercorrelations among subscales (*r* = 0.363 to 0.514) indicate that while these anxiety dimensions coexist within students’ experiences, each contributes unique variance, warranting separate assessment.

The empirical validation of EAIDAS addresses a critical gap identified in existing literature, which has predominantly examined students’ knowledge, attitudes, and perceptions of AI ethics without quantifying their emotional distress (Buele et al., 2025; Fošner, 2024; Acosta-Enriquez et al., 2024). The Academic Integrity Anxiety dimension validates concerns documented by researchers regarding plagiarism anxieties, unfair competitive advantages, and fear of false accusations from imperfect detection tools (Alawad et al., 2025; Nelson et al., 2025; Perkins et al., 2023). The Professional Future Anxiety subscale empirically captures the skill obsolescence concerns and career replaceability fears identified by Li et al. (2025) and Wang et al. (2025). The Societal Impact Anxiety dimension operationalizes students’ distress regarding complicity in bias perpetuation, privacy erosion, and environmental harm (Ashok et al., 2022; Goktas and Grzybowski, 2025), extending beyond previous studies that acknowledged these concerns without measuring their psychological burden.

The current exploratory and confirmatory factor analyses demonstrated strong alignment with the theoretically hypothesized three-dimensional structure of AI-related ethical anxiety. Parallel analysis empirically confirmed three distinct factors corresponding to Academic Integrity Anxiety, Professional Future Anxiety, and Societal Impact Anxiety, with both first-order and second-order models exhibiting excellent fit indices. This structural validation mirrors findings from other AI-related scales, where factor-analytic results consistently support theoretically proposed multidimensional frameworks (Maharmah et al., 2025; Alshaibani et al., 2025; Sallam et al., 2025). The three dimensions align closely with normative ethical frameworks underpinning EAIDAS development, specifically mapping onto principles of autonomy and accountability (Academic Integrity), justice and non-maleficence (Professional Future), and responsibility and fairness (Societal Impact) (Brendel et al., 2021; Nasir et al., 2024). This convergence between empirical structure and theoretical foundations strengthens the scale’s construct validity and ethical grounding.

The intercorrelations among EAIDAS dimensions reveal that ethical anxiety among university students is not fragmented but rather represents a coherent, future-oriented moral tension. The strongest correlation between Academic Integrity and Professional Future Anxiety (*r* = 0.514) suggests students perceive academic ethics as directly predictive of workplace conduct and employability, consistent with evidence linking academic dishonesty to unethical workplace behavior (Guerrero-Dib et al., 2020). The moderate correlations with Societal Impact Anxiety (*r* = 0.363 and *r* = 0.410) indicate that students moralize AI-related concerns at both the personal and systemic levels, linking individual conduct to broader risks, including inequity and cultural erosion (Buele and Llerena-Aguirre, 2025). This interconnected pattern mirrors documented technostress and ambivalence toward AI integration (Lin and Chen, 2024), in which perceived benefits and ethical risks create simultaneous psychological tensions across the domains of integrity, career, and society.

EAIDAS provides educators and institutional leaders with a validated diagnostic tool to identify students experiencing heightened ethical distress, enabling targeted interventions and support services. Universities can utilize EAIDAS data to inform policy development, ensuring AI guidelines address the specific integrity, career, and societal concerns generating student anxiety. The scale facilitates longitudinal monitoring of how institutional AI policies and educational interventions influence students’ ethical anxiety trajectories over time. Faculty can employ EAIDAS to assess the psychological impact of course-specific AI integration strategies, adjusting pedagogical approaches to mitigate unnecessary distress while maintaining academic standards. Mental health professionals in university counseling centers can use EAIDAS to screen for technology-related ethical anxiety and develop appropriate therapeutic interventions. The instrument also enables comparative research across institutions, disciplines, and cultural contexts, advancing our understanding of how educational environments differentially shape students’ ethical experiences with AI technologies.

Several methodological limitations warrant acknowledgment. The samples were drawn exclusively from Al-Azhar University in Egypt, specifically from two faculties in Dakahlia, limiting generalizability to students in different geographic regions, cultural contexts, and institutional environments with varying AI integration levels and ethical climates. Future studies should recruit participants from multiple universities and international contexts to establish cross-cultural measurement invariance. The cross-sectional design prevents causal inferences regarding how institutional policies, pedagogical practices, or individual AI usage patterns influence anxiety trajectories over time. The three-week test–retest interval, while adequate for assessing stability, may not capture longer-term fluctuations as AI technologies and institutional policies evolve. Self-report methodology introduces potential response biases including social desirability and retrospective recall limitations; future research should consider triangulating EAIDAS scores with behavioral measures to mitigate these concerns. The absence of predictive validity assessment limits understanding of how EAIDAS scores relate to actual academic performance and ethical decision-making outcomes. Finally, the scale development occurred during a period of rapid AI evolution, potentially rendering specific items less relevant as technologies and ethical discourse continue advancing.

Future validation studies should examine EAIDAS across diverse international samples, institutional types, and academic disciplines to establish cross-cultural measurement invariance and generalizability. Longitudinal research tracking students throughout their academic careers would illuminate how ethical AI anxiety evolves with increased exposure, changing institutional policies, and proximity to workforce entry. Researchers should investigate the scale’s criterion-related and predictive validity by examining relationships with behavioral outcomes including AI usage patterns, academic performance, and ethical decision-making in authentic academic scenarios. Studies should also systematically examine differential anxiety profiles across gender, academic year, and disciplinary background, as these variables may moderate relationships among EAIDAS dimensions. Studies exploring institutional factors associated with varying anxiety profiles would further inform targeted interventions. Experimental research evaluating how specific pedagogical interventions reduce EAIDAS scores would establish the scale’s sensitivity to change. Finally, qualitative and mixed-methods approaches complementing EAIDAS administration could provide deeper contextual understanding while helping triangulate self-report findings.

The EAIDAS is a theoretically grounded, psychometrically sound instrument that addresses a critical measurement gap in higher education research. EAIDAS successfully operationalizes the distinct psychological burden students experience when navigating ethical uncertainties surrounding AI integration in academic contexts. The scale’s robust three-dimensional structure, excellent reliability, and strong validity evidence position it as a valuable tool for researchers, educators, and policymakers seeking to understand and address students’ ethical distress. As artificial intelligence becomes increasingly embedded in educational environments, systematic measurement of students’ ethical anxiety becomes essential for developing supportive policies, effective interventions, and ethically responsible AI integration strategies. EAIDAS provides the empirical foundation necessary for evidence-based approaches to supporting students as they navigate the complex ethical landscape of AI-enhanced education, ultimately contributing to healthier, more ethically informed academic communities.

## Data Availability

The raw data supporting the conclusions of this article will be made available by the authors, without undue reservation.
