# Supplementary material for: Development and psychometric validation of the ethical AI Dilemma Anxiety Scale among university students
Source: Front Psychol. 2026 Apr 8;17:1810679. doi: 10.3389/fpsyg.2026.1810679 (PMC13099756; doi:10.3389/fpsyg.2026.1810679)
Supplement: Supplementary file 1 [file Table_1.docx]

**Appendix A**

*Ethical AI Dilemma Anxiety Scale (EAIDAS)*

**Instructions:** Please rate your level of agreement with each statement using the following scale:

**Response Options:** 1 = Strongly Disagree, 2 = Disagree, 3 = Neutral, 4 = Agree, 5 = Strongly Agree

Dimension 1: Academic Integrity Anxiety

1. I worry about ethical boundaries when using AI for assignments.
2. I feel anxious that peers using AI have unfair advantages.
3. I'm uncertain what AI use is acceptable in coursework.
4. I stress over whether AI-assisted work reflects my own learning.
5. I worry my genuine work will be flagged as AI-generated.
6. I feel anxious that AI devalues the skills I'm developing.
7. I feel conflicted by inconsistent AI policies across courses.
8. I'm unsure if using AI for brainstorming compromises integrity.

Dimension 2: Professional Future Anxiety

1. I worry AI will make my career field obsolete.
2. I fear the skills I'm learning will become irrelevant.
3. I stress about competing with AI for future jobs.
4. I'm uncertain which human skills will remain valuable.
5. I worry about ethical issues in AI-related industries.
6. I fear being pressured to use AI against my values at work.
7. I doubt my degree prepares me for an AI-transformed economy.
8. I worry about responsibility for AI harms in my profession.

Dimension 3: Societal Impact Anxiety

1. I worry about AI decisions affecting lives without oversight.
2. I fear AI will amplify biases and social inequalities.
3. I stress about privacy erosion from AI surveillance.
4. I worry AI misinformation will harm public discourse.
5. I fear humanity becoming too dependent on AI thinking.
6. I'm anxious about the environmental costs of AI systems.
7. I worry about AI power concentrating in few hands.
8. I feel burdened by my generation's responsibility for AI ethics.

**ملحق أ**

*مقياس قلق المعضلات الأخلاقية للذكاء الاصطناعي*

**تعليمات:** يرجى تحديد درجة موافقتك على كل عبارة وفقًا للمقياس التالي:

**خيارات الإجابة:** 1 = أرفض بشدة، 2 = أرفض، 3 = محايد، 4 = أوافق، 5 = أوافق بشدة

البعد الأول: القلق المتعلق بالنزاهة الأكاديمية

1. أشعر بالقلق من تجاوز الحدود الأخلاقية عند استخدام الذكاء الاصطناعي في واجباتي.
2. يقلقني أن زملائي الذين يستخدمون الذكاء الاصطناعي يحصلون على أفضلية غير عادلة.
3. أشعر بالحيرة حول ما هو مقبول وما هو مرفوض من استخدامات الذكاء الاصطناعي في دراستي.
4. أتوتر عندما أفكر فيما إذا كان عملي بمساعدة الذكاء الاصطناعي يعكس تعلمي الحقيقي.
5. يقلقني أن يُتهم عملي الأصيل بأنه من إنتاج الذكاء الاصطناعي.
6. أشعر بالقلق من أن الذكاء الاصطناعي يقلل من قيمة المهارات التي أسعى لاكتسابها.
7. أشعر بالارتباك بسبب اختلاف سياسات استخدام الذكاء الاصطناعي بين المقررات.
8. لا أعرف إن كان استخدام الذكاء الاصطناعي للعصف الذهني يخل بنزاهتي الأكاديمية.

البعد الثاني: القلق المتعلق بالمستقبل المهني

1. أخشى أن يجعل الذكاء الاصطناعي تخصصي المهني بلا جدوى.
2. أشعر بالقلق من أن المهارات التي أتعلمها ستصبح غير مطلوبة.
3. يقلقني التنافس مع الذكاء الاصطناعي على فرص العمل مستقبلاً.
4. أشعر بعدم اليقين حول المهارات البشرية التي ستظل ذات قيمة.
5. تؤرقني القضايا الأخلاقية المرتبطة بالعمل في مجالات الذكاء الاصطناعي.
6. أخشى أن أُجبر على استخدام الذكاء الاصطناعي بطرق تتعارض مع قيمي في عملي.
7. أشك في أن شهادتي الجامعية تؤهلني لسوق عمل يهيمن عليه الذكاء الاصطناعي.
8. يقلقني تحمل المسؤولية الناتجة عن أضرار الذكاء الاصطناعي في مهنتي مستقبلاً.

البعد الثالث: القلق المتعلق بالتأثير المجتمعي

1. يقلقني أن الذكاء الاصطناعي يتخذ قرارات تمس حياة الناس دون رقابة كافية.
2. أخشى أن يعزز الذكاء الاصطناعي التحيزات والفوارق الاجتماعية القائمة.
3. أشعر بالتوتر من تآكل الخصوصية بسبب أنظمة المراقبة الذكية.
4. يقلقني أن المعلومات المضللة المولدة بالذكاء الاصطناعي تضر بالحوار العام.
5. أخشى أن يصبح البشر معتمدين بشكل مفرط على الذكاء الاصطناعي في التفكير.
6. أشعر بالقلق من التكلفة البيئية لتشغيل أنظمة الذكاء الاصطناعي.
7. يقلقني تركز قوة الذكاء الاصطناعي في أيدي جهات محدودة.
8. أشعر بثقل المسؤولية الملقاة على جيلي في معالجة قضايا أخلاقيات الذكاء الاصطناعي.
